# Effect of Photoinitiator on Precursory Stability and Curing Depth of Thiol-Ene Clickable Gelatin

**DOI:** 10.3390/polym13111877

**Published:** 2021-06-05

**Authors:** Kai-Hung Yang, Gabriella Lindberg, Bram Soliman, Khoon Lim, Tim Woodfield, Roger J. Narayan

**Affiliations:** 1 Department of Materials Science & Engineering, North Carolina State University, Raleigh, NC 27965-7907, USA; kyang7@ncsu.edu; 2 Christchurch Regenerative Medicine and Tissue Engineering (CReaTE) Group, University of Otago, Christchurch 8011, New Zealand; gabriella.lindberg@otago.ac.nz (G.L.); bram.soliman@postgrad.otago.ac.nz (B.S.); khoon.lim@otago.ac.nz (K.L.); tim.woodfield@otago.ac.nz (T.W.); 3 Joint Department of Biomedical Engineering, University of North Carolina and North Carolina State University, Raleigh, NC 27695-7115, USA

**Keywords:** photoinitiator, hydrogel, polymerization, visible light, gelatin, thiol-ene click chemistry

## Abstract

Recent advances highlight the potential of photopolymerizable allylated gelatin (GelAGE) as a versatile hydrogel with highly tailorable properties. It is, however, unknown how different photoinitiating system affects the stability, gelation kinetics and curing depth of GelAGE. In this study, sol fraction, mass swelling ratio, mechanical properties, rheological properties, and curing depth were evaluated as a function of time with three photo-initiating systems: Irgacure 2959 (Ig2959; 320–500 nm), lithium phenyl-2,4,6-trimethylbenzoylphosphinate (LAP; 320–500 nm), and ruthenium/sodium persulfate (Ru/SPS; 400–500 nm). Results demonstrated that GelAGE precursory solutions mixed with either Ig2959 or LAP remained stable over time while the Ru/SPS system enabled the onset of controllable redox polymerization without irradiation during pre-incubation. Photo-polymerization using the Ru/SPS system was significantly faster (<5 s) compared to both Ig2959 (70 s) and LAP (50 s). Plus, The Ru/SPS system was capable of polymerizing a thick construct (8.88 ± 0.94 mm), while Ig2959 (1.62 ± 0.49 mm) initiated hydrogels displayed poor penetration depth with LAP (7.38 ± 2.13 mm) in between. These results thus support the use of the visible light based Ru/SPS photo-initiator for constructs requiring rapid gelation and a good curing depth while Ig2959 or LAP can be applied for photo-polymerization of GelAGE materials requiring long-term incubation prior to application if UV is not a concern.

## 1. Introduction

Hydrogels are ideal materials for use as tissue engineering scaffolds as they are composed of hydrophilic polymer networks that hold a significant amount of water [1,2,3]. The highly hydrated nature and physicochemical properties of hydrogels resemble the native extracellular matrix (ECM), making these materials a promising choice for biomedical applications, especially with the growing interest in 3D cell-seeded structures over recent decades. Applications for hydrogels include cell-seeded scaffolds and 3D models for studying cellular behavior (e.g., models for cell growth in cancer) [4,5,6,7,8,9]. 

Gelatin is one of the most studied biomaterials as it is well characterized, biodegradable, and water-soluble [10]. However, pure gelatin exhibits a low gelation temperature that behaves as liquid above 30 °C; as such, it cannot be used for cell culture at physiological temperature (37 °C). To overcome this limitation, gelatin is often conjugated with functional groups that can then facilitate interchain crosslinking via free radical chain-growth polymerization [11,12]. Our recent report highlights that a thiol-ene crosslinkable gelatin, allylated gelatin (GelAGE), can also be used to fabricate tissue engineering scaffolds that exhibit appropriate mechanical strength and shape retention to support cell growth, cell proliferation [13], and withstand erosion by the external environment. It has been suggested that thiol-ene based step-growth polymerization systems, in general, are less susceptible to oxygen inhibition compared to more commonly applied vinyl chain-growth systems [14]. This parameter may pose a major advantage when it comes to fabricating hydrogel scaffolds that can act as a structural 3D support for cells, be fashioned into clinically relevant dimensions for personalized medicine applications, and be easily handled in a clinical setting. In addition to choosing an appropriate crosslinking strategy, the employment of light to trigger photocrosslinking has been widely exploited, which permits spatiotemporal control over material reaction and rapid fabrication [15]. It has further been observed that the selection of photo-initiator platforms plays a crucial role in limiting oxygen inhibition under ambient atmosphere. Detailed optimization of each biomaterial system is necessary to obtain appropriate structural stability for gelatin-based materials [16]. 

To date, a number of photoinitiators absorbing photons from the UV region to the visible light region have been investigated for polymerization of gelatin-based biomaterials. Lithium phenyl-2,4,6-trimethylbenzoylphosphinate (LAP) and 2-hydroxy-1-[4-(hydroxyethoxy)-phenyl]-2-methyl-1-propanone (Ig2959) are commonly applied type I photoinitiators, which undergo photocleavage to create free radicals [17,18,19]. These photoinitiators absorb light in the UVA range (320–400 nm); LAP also exhibits absorption in a narrow visible light range (400–420 nm). However, a drawback to polymerizing with UV light is the possibility of genotoxicity [20,21] and weakening of cell membrane integrity associated with exposure to reactive oxygen species (ROS) [22]. Additionally, UV light may be attenuated by tissue [23]; this issue may limit the use of UV polymerization of hydrogels for tissue engineering applications as it may not be possible to fabricate clinically relevant sized samples in vivo due to limited penetration depth [24,25,26]. Still, LAP is commonly used in both the UVA [18] and visible light range [17] to maximize absorption and crosslinking efficiency due to its low absorptivity in the visible light range. In recent years, our team has conducted studies of another visible light photoinitiator system consisting of tris-bipyridyl ruthenium (II) hexahydrate (Ru) and sodium persulfate (SPS), which is capable of initiating polymerization of gelatin-based hydrogels, with high shape fidelity, cell viability, and cell metabolic activity; this system demonstrated a lower level of oxygen inhibition than conventional type I photoinitiators [13,16,17,27]. Due to reduced oxygen inhibition when applying the recyclable Ru/SPS system, it has specifically been revealed that Ru/SPS can be used to fabricate vinyl functionalized gelatin (GelMA) scaffolds with significantly greater light penetration depth as compared to the UV-initated Ig2959 photo-initiator system. The choice of photo-initiator platform is evidently necessary to be optimised in order to control the fabrication window and thus clinical applicability of any photo-polymerisable material platform.

While promising results have been seen with chain-growth mechanism [16,17], it has not yet been studied how specific photo-initiator systems may affect the light-curing depth of thiol-ene clickable hydrogels to fabricate large scale constructs. Likewise, considering prolonged surgical procedures, the stability of both end point property and gelation kinetics of GelAGE precursory solution has not been reported, although it can largely affect the reliability of resultant constructs and overall fabrication time. Hence, the aim of this study was to systematically explore the stability of the precursory solution, gelation kinetics, and curing depth of GelAGE incubated with three distinct, commonly applied photoinitiators: Ru/SPS, LAP, and Ig2959. The replenishment of photoinitiators was further investigated as a strategy to modulate physicochemical properties and gelation kinetics following longer periods of pre-incubation. In this approach, we harness the power of different photo-initiator systems to overcome limited fabrication windows, handleability issues, heterogeneous network formation, as well as restricted sample height.

## 2. Materials and Methods 

GelAGE was synthesized as described in the literature [13]. In brief, gelatin (porcine skin, type A) was first dissolved in milli-Q water to 10 wt% concentration and then reacted with 12 mmol of allyl glycidyl ether (AGE) and 2 mmol NaOH per gram of gelatin at 65 °C for 1 h to initiate fragmentation and modification. The resulting solution was adjusted to pH 7.4 using aqueous HCl, dialyzed with a cellulose membrane (MWCO 1 kDa Spectra/Por^®^ 7 Dialysis Tubing, Repligen, Rancho Dominguez, CA, USA) against milli-Q water, then lyophilized to isolate the GelAGE macromer. NMR spectra were collected on a Varian Unity INOVA 500 MHz spectrometer (Agilent Technologies, Santa Clara, CA, USA); the degree of modification (DoM) verified to be 0.79 mmol allyl/gram gelatin, indicating successful synthesis of this material. 

The macromer solution for crosslinking was composed of 20 wt% lyophilized GelAGE in PBS and 60 mM dithiothreitol (DTT) for a final 1:0.75 AGE:SH molar ratio as well as one of the following three photoinitiators: (1) 1 mM Ru combined with 5, 10, or 20 mM sodium persulfate (SPS), (2) 0.05 wt% LAP, or (3) 0.05 wt% Ig2959. All the photoinitiators were first dissolved in DI water and added to the macromer solution with a specific *v/v* ratio to achieve the final concentration. The photoinitiator formulation and concentrations are listed in Table 1.

To study mass loss, mass swelling ratio, compressive modulus, depth of cure, and curing volume, cylindrical hydrogel discs were cast by transferring macromer solutions into silicone molds (5.5 mm diameter, 2 mm depth), pre-incubating for either 0 or up to 30 min, then crosslinking with 3 min exposure at 30 mW/cm^2^ light intensity (OmniCure S1500, 320–500 nm light guide, Excelitas Technologies, Waltham, MA, USA); a 400–450 nm Rosco IR/UV filter equipped to OmniCure^®^ was used with samples containing Ru/SPS to enable visible light crosslinking. Details on the light intensity measurements can be found as Supplementary Information. The light filter was removed while crosslinking with samples containing LAP and Ig2959. All of the experiments were performed under ambient conditions without nitrogen purging. The initial mass was obtained immediately after photopolymerization (*m*_initial_); the mass after 24 h incubation in PBS (*m*_swelling_) was also noted. The mass after lyophilization of swollen discs (*m*_dry, swelling_) and non-swollen discs (*m*_dry, non- swelling_) was noted; the *m*_initial, dry_, sol fraction, and swelling ratio(q) were calculated as follows:

Actual macromer fraction = *m*_dry, non-swelling_/*m*_initial_ (1)

*m*_initial, dry_ = *m*_initial_ × actual macromer fraction (2)

sol fraction = (*m*_initial, dry_ − *m*_dry, swelling_)/*m*_initial, dry_ (3)

*q* = *m*_swelling/_*m*_dry, swelling_ (4)

Compression testing of swollen hydrogel discs (24 h incubation at 37 °C in PBS) was performed on an MTS Criterion^®^ Series 40 Model 42 instrument (MTS, Murfreesboro, TN, USA) with uniaxial single compression at a speed of 0.01 mm/s and a preload value of 0.015 N. Compressive modulus data was obtained by fitting the stress-strain curve at a 10–15% strain range; stress-strain curves for all of the samples were linear within this range.

The macromer solution exposed to 3 min of 30 mW/cm^2^ light within a silicone mold with a deep cylindrical channel (5.5 mm diameter, h = 10 mm) was used to study the depth of cure by measuring the resultant height and diameter of the hydrogel with respect to the depth of the mold.

Rheology measurement was performed with a plate-plate (steel and glass) geometry at 20 °C, 0.2 mm gap, and 489.9 mm^2^ contact area (Physica MCR 301 rheometer, Anton Paar, Graz, Austria) with a solvent trap to reduce drying of the macromer solution during the measurements. Oscillation measurements at 0.1% strain and 1 Hz conditions within the linear viscoelastic range as determined by amplitude sweep were performed; the storage and loss moduli with respect to time were monitored. All of the experiments were performed under a light-protective hood to remove any interference from ambient light. A macromer solution (150 µL) was transferred to the glass plate and exposed to visible light or UV light from the bottom side of the glass plate with the same intensity as the cast gel. The time to gelation was the indicator of gelation kinetics which was defined as the time required from initiation of irradiation to the time point of storage modulus and loss modulus cross over. For instant crosslinking, macromer solutions were added onto the glass plate and subjected to oscillation for 1 min to reach steady-state prior to in situ measurements of crosslinking. Crosslinking with pre-incubation was conducted with the mixed macromer solution incubating in a microtube, which was covered with aluminum foil to protect from light; the data was compared with pre-incubation under shearing for 1800 s. Comparisons were made to polymers with and without replenishment of photoinitiators (SPS for the Ru/SPS formulation, LAP for the LAP formulation, and Ig2959 for the Ig2959 formulation) after 30 min pre-incubation by adding the same amount as in the precursory solution. For convenience, curves with only storage modulus were shown for facile identification. 

Statistical analysis of replicates conducted using GraphPad Prism 8.4.1 (GraphPad Software, San Diego, CA, USA). The comparison of sol fraction, swelling ratio, and compressive modulus between time points of pre-incubation (same group) was conducted through one-way ANOVA with the Tukey post-hoc test; the comparison between samples before and after replenishment (different group) was conducted through the *t* test. It should be noted that the number of replicates is N = 9 except the data points of non-measurable and no-gel formation. Depth of cure with different photo-initiators was also compared using one-way ANOVA with the Tukey post-hoc test with N = 6. The results were deemed statistically significant for *p* < 0.05.

## 3. Results

### 3.1. Sol Fraction and Swelling Ratio

To evaluate the differences in network composition as well as the stability of crosslinking as a function of time, the physicochemical properties and mechanical strength of the hydrogel constructs were analyzed. The sol fraction was defined as the percentage of macromers not crosslinked within the polymer network and was used as a measure to reflect the crosslinking efficiency. Similarly, the swelling ratio represents the amount of water taken up by the hydrogel, reflecting the interchain spacing of the network, and was calculated as the fractional increase in the weight of the hydrogel. The characteristics of the constituent polymer influence the mechanical properties of the bulk hydrogel construct; these physical cues that are known to affect the development of seeded cells. Precursory solutions with commonly used photo-initiator formulations (Ru/SPS, Ig2959 and LAP) were prepared. 

**Figure 1:**
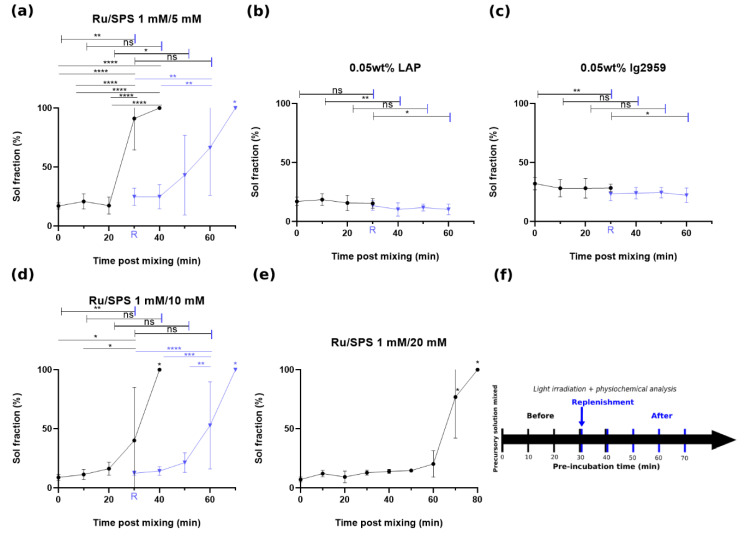
Sol fraction of GelAGE versus pre-incubation time prior to crosslinking with flow chart, circle (•) represents crosslinking with designated time of pre-incubation and triangle (▼) represents the samples with further pre-incubation after replenishment of photoinitiator at t = 30 min: (**a**) Sol fraction: 1 mM/5 mM Ru/SPS, (**b**) Sol fraction: LAP, (**c**) Sol fraction: Ig2959, (**d**) Sol fraction: 1 mM/10 mM Ru/SPS, (**e**) Sol fraction: 1 mM/20 mM Ru/SPS and (**f**) flow chart of the experiment. Values in the figures represent means and standard deviation of N = 9 replicates. Capped lines are for comparison with different groups (before and after replenishment). Asterisks (*) represents values statistically significant at the *p* < 0.05 level, ** for *p* < 0.01, *** for *p* < 0.001 and **** for *p* < 0.0001. ns represents values statistically non-signigicant.

**Figure 2:**
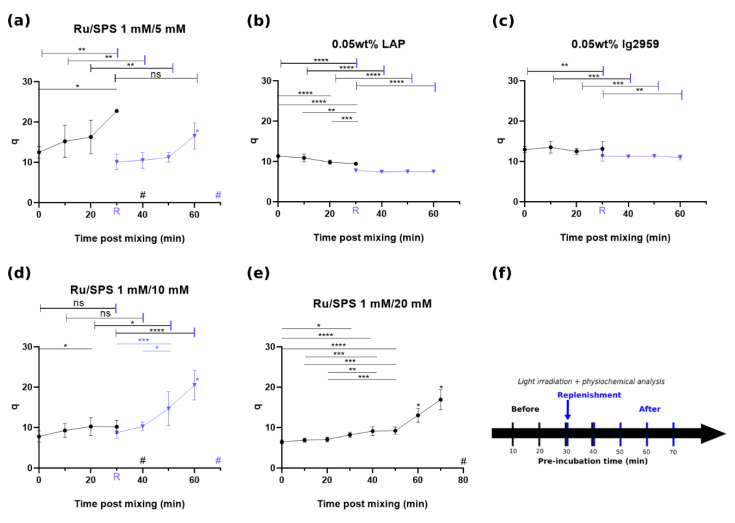
Mass swelling ratio of GelAGE versus pre-incubation time prior to crosslinking with flow chart, circle (*•*) represents crosslinking with designated time of pre-incubation and triangle (▼) represents the samples with further pre-incubation after replenishment of photoinitiator at t = 30 min: (**a**) q: 1 mM/5 mM Ru/SPS, (**b**) q: LAP, (**c**) q: Ig2959, (**d**) q: 1 mM/10 mM Ru/SPS and (**e**) q: 1 mM/20 mM Ru/SPS and (**f**) flow chart of experiment. Values in the figures represent mean and standard deviation of N = 9 replicates. Capped lines are for comparison with different groups (before and after replenishment) while straight lines are for comparison within the same group. Asterisks (*) represents values statistically significant at the *p* < 0.05 level, ** for *p* < 0.01, *** for *p* < 0.001 and **** for *p* < 0.0001. Pound(#) sign represents no-gel formation. ns represents values statistically non-signigicant.

As demonstrated in Figure 1a–c, the sol fraction of casted gels with 1 mM Ru with 5 mM SPS, LAP and Ig2959 were 16.9 ± 2.4, 16.9 ± 3.7 and 32.1 ± 5.3%, respectively, following instant irradiation (t = 0). The corresponding mass swelling ratio (*q* values) were 12.5 ± 1.5, 11.4 ± 0.4 and 13 ± 0.8 as seen in Figure 2a–c. For these three formulations, casted gel with Ig2959 exhibited the highest sol fraction and swelling ratio. To understand the stability of these photoinitiator formulations, the sol fraction and q value with different pre-incubation time was recorded. For these three conditions, statistical analysis showed significant difference between instant crosslinking and after 30 min incubation for 1 mM/5 mM Ru/SPS and q for LAP. In general, casted gels crosslinked with Ru/SPS demonstrated a noteworthy change in sol fraction and swelling ratio while values for LAP and Ig2959 crosslinked gel remained more stable and stayed in a similar range following pre-incubation. It was furthermore noted that after 30 min of pre-incubation, macromer solutions photocrosslinked with 1 mM/5 mMRu/SPS were unable to crosslink. This inability to form a gel is labeled as 100% sol fraction in Figure 1 and a pound sign (#) representing no-gel formation in Figure 2. It is believed that SPS was depleted in the macromer solution via redox crosslinking [28], which resulted in insufficient crosslinking later when irradiation was applied. Increasing the initial concentration of SPS to 10 mM and 20 mM was able to enhance the stability; the changes in sol fraction and swelling ratio between instant crosslinking and crosslinking after 30 min pre-incubation were decreased. Although the pre-incubation time associated with a compromise in the ability to gel was the same for gels containing 10 mM and 5 mM SPS, the sol fraction and swelling ratio were much lower for gels containing 10 mM SPS after 30 min pre-incubation. By further increasing the SPS concentration to 20 mM, it took macromers around 70 min to become incapable of photocrosslinking, which was approximately double the time as compared to lower SPS concentrations. This result indicated that the phenomenon appeared to be a time-dependent process and driving force for depletion was insufficient for consuming this high concentration of SPS in 30 min. However, this high concentration of initial SPS made macromers undergo partial gelation without irradiation. Again, this result may be attributed to redox-crosslinking by SPS and DTT before the introduction of irradiation and Ru radicals [28].

In addition to increasing the initial SPS concentration, replenishment of SPS after pre-incubation was found to enable photo-crosslinking following prolonged incubation times. A flow chart of pre-incubation and time for replenishment is shown in Figure 1f. This replenishment of SPS resulted in a decrease of both sol fraction and q value; an increase in both values was noted with further incubation. A statistical comparison between before and after replenishment is indicated by the capped line. Since using 20 mM SPS caused notable GelAGE redox crosslinking in the absence of light, mixing additional SPS for replenishment was not a viable strategy when using this formulation. On the other hand, the replenishment of LAP or Ig2959 resulted in an overall decrease in the sol fraction and q value. Since the crosslinking efficiency and interchain spacing were closely correlated with mechanical strength, the results were compared with the compressive modulus values obtained by uniaxial single compression. 

### 3.2. Mechanical Testing

**Figure 3:**
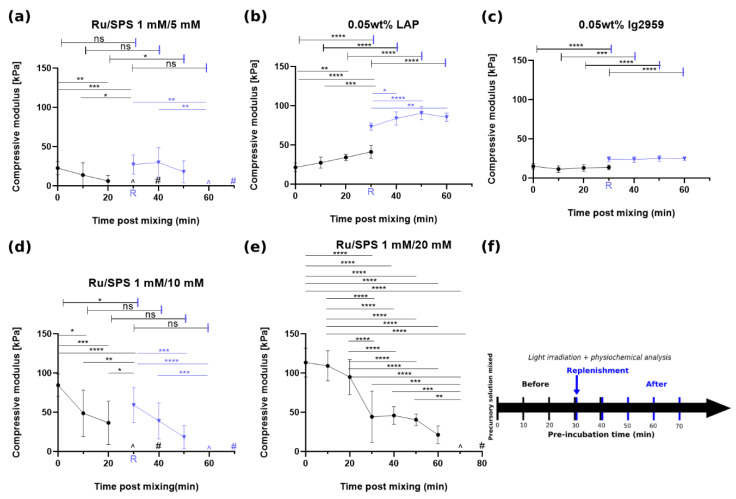
Mechanical strength of GelAGE versus pre-incubation time prior to crosslinking with flow chart, circle (*•*) represents crosslinking with designated time of pre-incubation and triangle (▼) represents the samples with further pre-incubation after replenishment of photoinitiator at t = 30 min: (**a**) 1 mM/5 mM Ru/SPS, (**b**) LAP, (**c**) Ig2959, (**d**) 1 mM/10 mM Ru/SPS and (**e**) 1 mM/20 mM Ru/SPS and (**f**) flow chart of the experiment. Values in the figures represent means and standard deviation of N = 9 replicates. Capped lines are for comparison with different groups (before and after replenishment) while straight lines are for comparison within the same group. Asterisks (*) represents values statistically significant at the *p* < 0.05 level, ** for *p* < 0.01, *** for *p* < 0.001 and **** for *p* < 0.0001. Pound (#) sign represents no-gel formation and (^) represents non-measurable. ns represents values statistically non-signigicant.

For GelAGE crosslinked with 1 mM/5 mM Ru/SPS, a reduction in compressive modulus was observed as a function of pre-incubation time, dropping from 22.7 ± 8.4 kPa for instant crosslinking to being as low as “non-measurable” (<2 kPa) after 30 min pre-incubation and the absence of gel formation after 40 min (as shown in Figure 3a). 

Compared with gels crosslinked with LAP or Ig2959, the resultant compress modulus values were 21.4 ± 5.6 kPa and 15 ± 3.4 kPa, respectively, with no observation of incapability of crosslinking after pre-incubation. Interestingly, the compressive modulus of GelAGE crosslinked with LAP was observed to instead increased with pre-incubation, reaching 41.2 ± 3.8 kPa after 30 min pre-incubation, which may indicate ongoing dark polymerization in this experiment as stray light may initiate the crosslinking during pre-incubation [29]. Unlike the other crosslinked gels, GelAGE crosslinked with Ig2959 exhibited stable mechanical strength spanning across the full 30 min pre-incubation time, with no significant difference detected (*p* > 0.05). This modulation of compressive modulus with pre-incubation time has not been previously reported in the literature. It is speculated that this phenomenon results from the depletion of SPS in the macromer solution instead of from the depletion of Ru since Ru is known to be recyclable in the type II co-initiator system [17]. Furthermore, the compressive modulus after replenishment returned to at least 70% of the value associated with instant crosslinking. This result rules out the depletion of the DTT crosslinker in the macromer solution. Similar to sol fraction results, an increase in initial SPS concentration was able to prolong the stability of macromer solution, which enabled crosslinking; the gels exhibited compressive modulus values of 84.5 ± 14.3 kPa with 10 mM SPS and 113.5 ± 18.5 kPa with 20 mM SPS. In the case of 20 mM SPS, it took 70 min to observe non-measurable gels with partial gelation. Again, by replenishing SPS after pre-incubation and regaining crosslinking capability, subsequent photocrosslinking resulted in cast gels with compressive modulus values of 27.2 ± 12.4 kPa and 59 ± 22.6 kPa (30 min pre-incubation + SPS) for 5 mM and 10 mM SPS, respectively; these results stand in strong contrast to the non-measurable gels (30 min pre-incubation) that were obtained without replenishment. Then macromer solution gradually loses the capability of crosslinking with further pre-incubation again; hence, lower mechanical strength values were noted. In contrast, the replenishment of LAP resulted in a further increase in compressive modulus to 73.4 ± 3.3 kPa. The compressive modulus reached 85.4 ± 5.8 kPa after another 30 min pre-incubation. Replenishment of Ig2959 increased the compressive modulus value since the concentration of photoinitiator was doubled; this phenomenon has been previously observed [29,30]. 

### 3.3. Rheological Testing

Although mechanical, sol fraction, and q measurements provided the information on the effectiveness and outcome of crosslinking, these measurements serve as end point measurements of the polymerization process. Thus, interest was focused on obtaining continuous and in situ measurements. Rheological testing is furthermore important for downstream applications such as extrusion-based printing as well as lithography-based printing since the rheological properties of the material determine the elastic and viscous behavior of the material and handleability in constructs with clinically relevant size. The storage modulus and loss modulus represent the elastic and viscous components of the material. These parameters were tracked in real time via photo-rheological measurements. 

**Figure 4:**
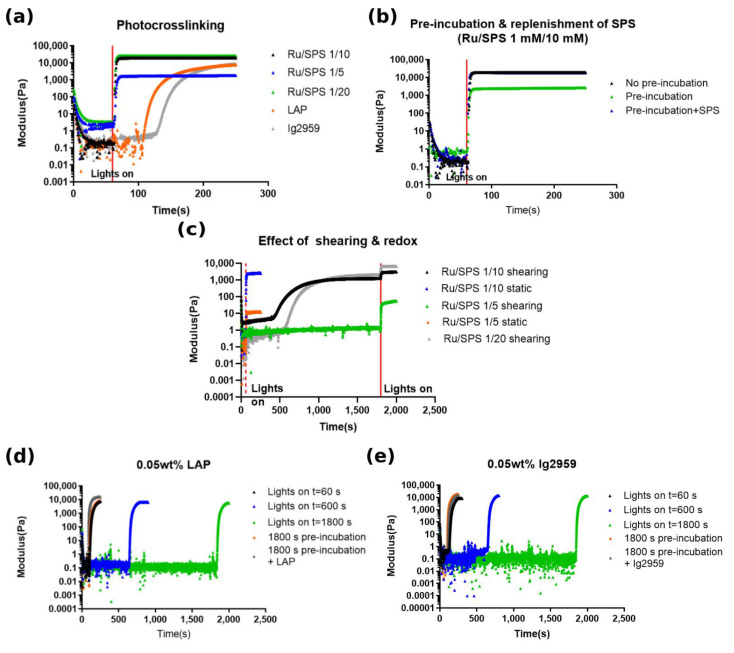
Rheological testing with photocrosslinking: (**a**) variation of storage modulus under irradiation after 60 s stabilization for all the five photoinitiator formulation, (**b**) variation of storage modulus with 1800 s static pre-incubation with and without replenishment of SPS (1 mM/10 mM Ru/SPS), (**c**) compare variation of storage modulus between the condition of 1800 s shearing and 1800 s static pre-incubation (red dash line represents the time to turn of the light for static samples and red solid line represents time to turn of the light for shearing samples), (**d**) variation of storage modulus for LAP under irradiation at different time points and effect of pre-incubation and replenishment of the photoinitiator and (**e**) variation of storage modulus for Ig2959 under irradiation at different time points and effect of pre-incubation and replenishment of the photoinitiator. Note the 1800 s static pre-incubation was performed in the microtube before applied to the rheometer.

In Figure 4a, the transition in storage modulus was compared among the five testing conditions with no pre-incubation. All of the Ru/SPS formulations crosslinked immediately upon light exposure; the LAP and Irgacure 2959 formulations exhibited a delay between light exposure and a marked increase in storage modulus. For GelAGE crosslinked with Ru/SPS, a lower initial SPS concentration resulted in a lower storage modulus value. All of the Ru/SPS formulations exhibited an immediate response to irradiation, showing a time to gelation of approximately 3 s. The modulus reached a plateau within another 4 s and remained constant until the end of the irradiation period. However, GelAGE crosslinked with LAP and Ig2959 demonstrated a slower gelation kinetics, with Ig2959 requiring the longest time. GelAGE crosslinked with LAP and Ig2959 took approximately 50 s and 70 s to reach the cross-over point, respectively, as shown in Table 2. It was observed that after the gel point was reached, the storage modulus continued to increase gradually with light exposure and ultimately stop after the irradiation switched off (3 min after the onset of exposure), which showed no dark polymerization [29]. 

Pre-incubation resulted in a lower storage modulus for the GelAGE crosslinked with Ru/SPS, while replenishment of SPS increased the storage modulus to the same level as the material without pre-incubation (Figure 4b). The gelation kinetics was not affected by pre-incubation since Ru, which interacts with the photons, was not depleted. Pre-incubation under shearing was also investigated to evaluate the end point storage modulus. 

In Figure 4c, an increase of modulus before irradiation was observed after 400–500 s shearing for GelAGE crosslinked with 1 mM/10 mM and 1 mM/20 mM Ru/SPS yielding a storage modulus around 100 times the value of loss modulus before irradiation. Interestingly, an increase in modulus was not observed for GelAGE crosslinked with 1 mM/5 mM Ru/SPS. It was further noted that both approaches for pre-incubation, placement in microtube or under shearing in the rheometer, exhibited similar storage moduli after irradiation.

Data from GelAGE crosslinked with LAP and Ig2959 are shown in Figure 4d,e, respectively. The results indicate no distinct change in storage modulus during pre-incubation. The time to gelation was not affected by pre-incubation. However, after adding 0.05 wt% photoinitiator again, the time to gelation was shortened from 50 s to 25 s for LAP and from 70 s to 40 s for Ig2959, indicating that the polymerization rate was concentration-dependent which was similar to concentration-dependent polymerization mechanism observed for chain growth systems [30].

### 3.4. Depth of Cure 

**Figure 5:**
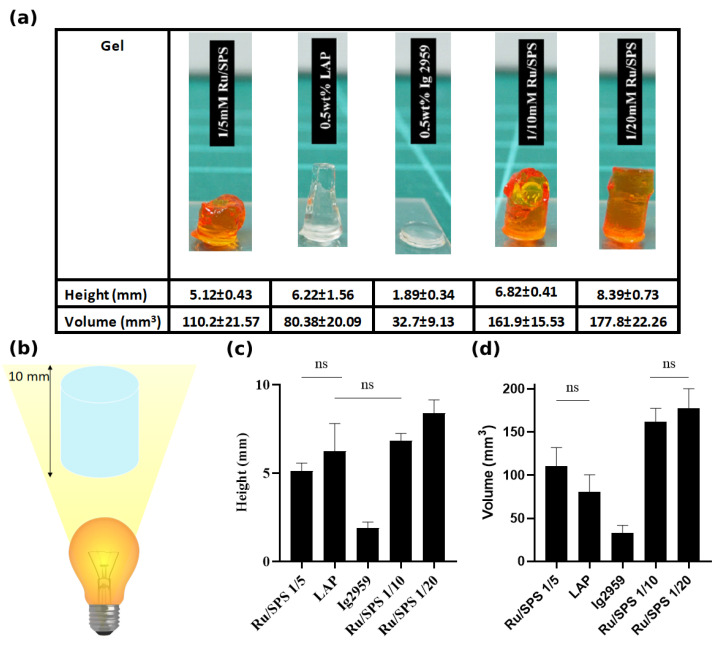
(**a**) Image of GelAGE cast in 10 mm mold with different photoinitiators showing distinct heights and volumes, (**b**) schematic figure showing the direction of light and designed shape of gel, (**c**) depth of cure, and (**d**) curing volume. Values in the figures represent means and standard deviation of N = 6 replicates. ns represents values statistically non-signigicant.

In addition to physicochemical properties and rheological behavior, the depth of cure is another parameter that affects the downstream applicability, including the utility for preparing constructs with clinically relevant size or applicability in injectable hydrogel systems. As such, the depth of cure was investigated via the height of cast gel with respect to the mold. Our results showed that macromers with 1 mM/20 mM Ru/SPS resulted in 8.39 ± 0.73 mm with almost full height and the highest curing volume of 177.8 ± 22.6 mm^3^ when applying a 10 mm deep mold, as shown in Figure 5a. 

GelAGE crosslinked with 1 mM/ 10 mM Ru/SPS and LAP results in gels with medium height, 6.82 ± 0.41 mm and 6.22 ± 1.56 mm, respectively; no significant difference between these measurements was noted. However, the cross-sectional area of the GelAGE cylinder crosslinked with LAP became lower from the exposed surface to the bottom of the gel, indicating less effective crosslinking at positions further away from the light source and showing half the curing volume compared with 1 mM/10 mM Ru/SPS. For gels with 1 mM/5 mM Ru/SPS, the depth of cure and curing volume exhibited no significant difference with gels with LAP but showed significant difference with 1 mM/10 mM Ru/SPS crossklinked gels. In contrast to the aforementioned formulations, GelAGE crosslinked with Ig2959 showed the lowest depth of cure and curing volume of 1.89 ± 0.34 mm and 32.7 ± 9.13 mm^3^. 

## 4. Discussion

GelAGE is a promising biomaterial due to its resistance to oxygen inhibition, formation of a homogeneous network by thiol-ene crosslinking, flexibility for adjusting material properties, and compatibility with photocrosslinking by photoinitiators that absorb UV or visible light. The stability and time-dependent physicochemical properties of GelAGE based on different photoinitiators observed in this study have not been reported in previous studies. On the exposure to UV light, GelAGE crosslinked with Ig2959 created structures with relatively low strength. The limitations associated with Ig2959 include low water solubility and, more importantly, cytotoxicity which has previously been reported to result in an upper limit to the Ig2959 concentration [18]. Furthermore, there are concerns related to the detrimental effect of UV exposure to cells embedded in hydrogel. 

Although the water solubility of LAP is up to 8.5 wt%, LAP has been reported to exhibited low molar absorptivity in a narrow visible light range (ε ≈ 30 M^−1^ cm^−1^ at 405 nm, 0.05 cm^−1^ absorptivity with 0.05 wt%) [18]. This was not enough to form gels using the GelAGE system with visible light initiation. The alternative absorption peak for LAP occurs at 375 nm (ε ≈ 220 M^−1^ cm^−1^, 0.36 cm^−1^ absorptivity with 0.05 wt%). Thus, a light source with wavelength spanning from UVA to the visible light range was exploited instead in this study to carry out successful gelation. GelAGE crosslinked with LAP exhibited moderate mechanical strength; no depletion of photoinitiator in precursory solution was observed during pre-incubation. Instead, an increase in mechanical strength was observed during pre-incubation and could result from dark polymerization with stray light in this experiment while sol fraction stayed at a similar range. Since the degree of modification was not measured in this study, the sol fraction may not directly reflect the mechanical strength. It’s possible to have the same amount of polymer chain diffuse out while having more crosslinking site and results in higher mechanical strength.

The co-initiator system with Ru and SPS was reported to have an extended absorption into the visible light range with high molar absorptivity (ε ≈ 14,600 M^−1^ cm^−1^ at 450 nm, 14.6 cm^−1^ absorptivity with 1 mM) [31]. The tailorability of compressive modulus with SPS concentration and pre-incubation provides a versatile strategy to achieve a range of mechanical properties. Although an increase in SPS concentration could extend the stability of the precursory solution from 30 min up to 70 min, concomitantly the redox crosslinking becomes strong enough to form a gel without the exposure of light. For this partial gelation, it was hypothesized that thiyl radicals were generated via a redox reaction between DTT and SPS, driving the reaction towards polymerization even without irradiation when higher initial levels of SPS were present. However, the lower SPS concentrations investigated in this study (5–10 mM) did not lead to partial gelation of the GelAGE precursory solution. As thiyl radicals are known to also form disulfide bonds [32], it is speculated that not all redox-generated thiyl radicals contribute to crosslinking GelAGE and a higher amount of SPS is thus required to yield gelation of the samples without any light exposure. Subsequently, when Ru participated in the reaction via light exposure, mitigation of disulfide bond formation occurred, thiyl radicals were regenerated, and polymerization overwhelmed as a consequence of additional thiol-ene crosslinking [29]. This phenomena may be useful to e.g., increase printability [28]. Thus, replenishment of SPS provides a strategy to modulate the properties for applications that require long periods of incubation while maintaining a liquid precursory solution. These two methods combined create a pathway for GelAGE as a promising candidate for the formation of constructs with mechanical gradients, which can serve as physical cues to facilitate guided proliferation, adhesion, migration, and differentiation of cells [19,33,34,35,36]. Also, control over the degree of crosslinking and interchain spacing as shown in Figure 1 and Figure 2 offer an opportunity for optimization of cell proliferation within the hydrogel. 

Rheological testing demonstrated rapid gelation kinetics for Ru/SPS formulation, which was independent of SPS concentration. This result indicated that regeneration of the Ru radical [17], as proposed by Lim et al., was rapid enough to react with SPS with a concentration at least twenty times higher. This rapid reaction can allow the overall process to be shortened with only a few seconds of irradiation for each step. However, a change in modulus during shearing was observed and seemed to depend on SPS concentration. One hypothesis is that this result is attributed to physical crosslinking by shearing with the aid of redox crosslinking via spontaneous reactions between DTT and SPS. For 1/10 mM and 1/20 mM Ru/SPS, this mild redox crosslinking could enable the subsequent physical crosslinking, thus require a prolonged time, and trigger the transition of macromer solution from liquid to gel-state during shearing. The oscillation during measurement could possibly accelerate the redox reaction and thus, while incubating in microtube, a low level of storage modulus for 10 m M SPS samples was observed before irradiation. In GelAGE with 1 mM/5 mM Ru/SPS, redox crosslinking is thought to be relatively minor due to lower SPS concentration; thus, no increase in modulus is observed in our result. Taken together with the physicochemical results, it clearly demonstrated that altering the SPS concentration can be utilized as a strategy to alter the viscosity and degree of redox crosslinking, which opened the operational window for printability of this material. Although a slower gelation kinetics and lag time [37] was observed for GelAGE crosslinked by LAP and Ig2959 due to radical scavenging [16] by the presence of oxygen; since these photoinitiators are not as recyclable as compared to Ru, a higher concentration of photoinitiator was capable of modulating the gelation kinetics. Recently, Holmes et al. demonstrated a thiol-ene photo-click hydrogel based on thiol-functionalized type-I collagen with UV light (365 nm) and LAP or Ig2959 as photoinitiator [38]. A variation in the time to complete gelation with respect to the concentration of photoinitiator was demonstrated. It should, however, be noted that a higher concentration of 0.1–0.5% (w/v) of LAP or Ig2959 was used and a lower light intensity of 4.45 mW/cm^2^ was applied as compared with our study. Compared to the Ru/SPS platform, the properties of GelAGE crosslinked with LAP or Ig2959 were further demonstrated to be stable over time. However, the required time to reach gelation may constrain the use of the materials and be incompatible with building clinically relevant constructs. Fairbanks et al. demonstrated a higher molar absorptivity and polymerization rate for LAP than for Ig2959 with the diacrylated poly(ethylene glycol) (PEGDA) hydrogel [18]. Our results were in accordance with the literature and further highlighted the superior polymerization rate that Ru/SPS can provide.

Although thiol-ene crosslinking is thought to provide a rapid reaction and Ru regenerates through a three-step cycle [17], a higher depth of cure was observed only for GelAGE crosslinked with 20 mM SPS. This result suggested that the three-step cycle could provide sufficient time for photons to reach a medium level(~5 mm) of the mold. However, it was speculated that a higher SPS concentration and secondary route of crosslinking by redox reaction, with no depth limitation, could be necessary to crosslink GelAGE with full height; in consequence, a lower depth of cure was observed for GelAGE crosslinked with 10 mM and 5 mM SPS. When comparing LAP and Ig2959, Fairbanks et al. reported that as LAP was photo-cleaved into radicals, the chromophores no longer existed, and light propagated more deeply into the cast gel. Materials crosslinked with Ig2959 have not been reported to exhibit such bleaching characteristics [18], resulting in extinction of chromophore after absorbing photons. Our results for LAP and Ig2959 were in accordance with these literature results as, for example, a low depth of cure for Ig2959 has previously been described. However, the low molar absorptivity in visible light range for LAP resulted in poor shape control, hindering the use of this material for fabricating bulky structures. The Ru/SPS platform, showing time-dependent viscosity and mechanical properties dependent on SPS concentration, may be a better choice to polymerize GelAGE when a higher depth of cure and more rapid gelation is required. On the other hand, LAP may be a better choice when a stable precursory solution is required and more straightforward due to the absence of redox crosslinking. However, UV light may still compromise the viability and generate gene toxicity. A recent report has shown that SPS-mediated redox could be exploited to controllably increase viscous properties in the absence of light while retaining the ability to fully photopolymerize remaining crosslinkable groups [28]. It is important to note that the synthesis route for GelAGE macromers is slightly different in our previous study, leading to lower molecular weight GelAGE macromers herein [13]. Macromer chain length could potentially influence the gelation kinetics and ability of redox-initiated thiyl radicals to achieve crosslinking rather than disulfide bond formation, which could result in different degrees of viscosity and mechanical strength.

## 5. Conclusions

In summary, we demonstrated successful polymerization of GelAGE with a range of visible light and UV light photoinitiators and observed distinct material properties after pre-incubation with different photoinitiator formulations based on the thiol-ene clickable reaction. For the visible light based Ru/SPS system, a wide range of material properties, including physicochemical properties, rheological properties, and depth of cure, can be obtained by controlling the SPS concentration or the pre-incubation time. Although the ability to crosslink is reduced following long-term pre-incubation, maintaining or recovery of the physiochemical properties was achieved by replenishment of SPS. This strategy allows the mitigation of any time-dependent variation and provides a viable option for applications that require long periods of incubation prior to crosslinking. It subsequently offers a strategy to prepare GelAGE constructs with uniform material properties. Compared to the widely adopted LAP and Ig2959 photoinitiators, Ru/SPS offered a facile approach for obtaining materials with tailorable physiochemical properties, rapid gelation kinetics, and tunable depth of cure using visible light. The required time to gelation may limit the use of LAP and Ig2959 for clinically relevant constructs. Especially, the low depth of cure for Ig2959 may preclude the use of this material for fabricating bulky structures. If a more stable precursory solution without redox crosslinking is required, LAP can be considered. However, the UV light may generate gene toxicity and compromise the viability for cells. 

## Figures and Tables

**Table 1:** Formulation of photoinitiators and irradiation condition to crosslink GelAGE.

**Hydrogel**	GelAGE				
**Photoinitiator**	Ru/SPS			LAP	Ig2959
**Concentration**	1 mM/5 mM	1 mM/10 mM	1 mM/20 mM	0.05 wt%	0.05 wt%
**Irradiation**	3 min, 30 mW/cm^2^				
**Wavelength**	400–500 nm			320–500 nm	

**Table 2:** Time to gelation(s).

**Photointiator**	Ru/SPS			LAP	Ig2959
**Concentration**	1 mM/5 mM	1 mM/10 mM	1 mM/20 mM	0.05 wt%	0.05 wt%
**Instant**	3	3.5	3	50	70
**Shearing** **(600 s)**	2	4	4	47	52.5
**Shearing** **(1800 s)**	3	4.5	5	42.5	51
**Pre-incubation (1800 s)**	3.5	3	-	41	51.5
**Pre-incubation + PI**	3	2	-	25	40

## Data Availability

The datasets generated during and/or analyzed during the current study are available from the corresponding author on reasonable request.

## Supplementary Materials

The following are available online at https://www.mdpi.com/article/10.3390/polym13111877/s1.
