# Supplementary material for: Effect of Photoinitiator on Precursory Stability and Curing Depth of Thiol-Ene Clickable Gelatin
Source: Polymers (Basel). 2021 Jun 5;13(11):1877. doi: 10.3390/polym13111877 (PMC8201352; doi:10.3390/polym13111877)

Supplementary Information

The intensity of exposure was measured both with and without filter to make sure the intensity is the same for both conditions so the position of the light source will be adjusted once the filter was adopted or removed. If the truncated cone was caused by non-parallel rays, we might be able to observe different geometry of the cone when placing the mold away from the center of the light exposure. However, no apparent difference of the geometry was observed which may indicate this effect is minor or can be neglected. Also, the opening of the light guide is wider compared with the diameter of the gel which can also alleviate the effect of divergence of rays.

The left image shows that the light only partially is able to penetrate the mould. However, very little light actually penetrates the mould (see graph). We tested this through measuring the light intensity through the mould and comparing these values to the light intensity when removing the mould (i.e., keeping the distance of the sensor to the light the same).

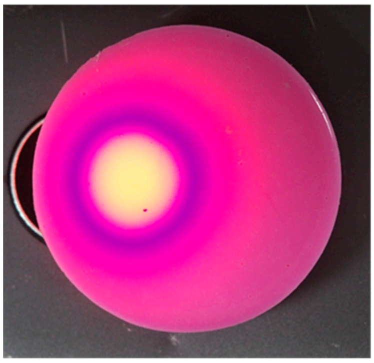

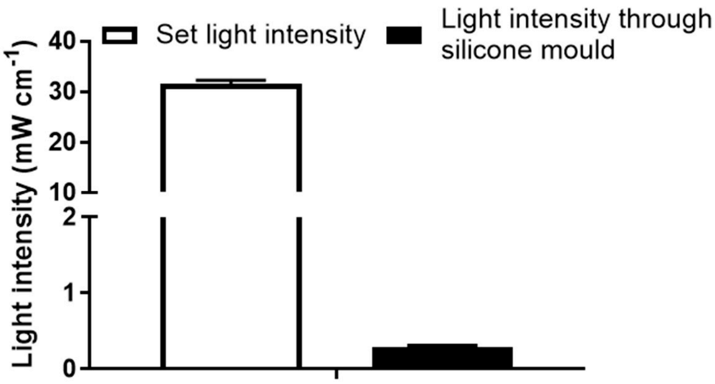

Supplement: Supplementary file 1 [file polymers-13-01877-s001.zip › polymers-1189424-SI.pdf]
